# Changes of head circumference and ventricular width in infant hydrocephalus managed with adjustable shunt valves and gravitational assistance—a comparative bi-center study

**DOI:** 10.1007/s00381-025-07074-y

**Published:** 2025-12-17

**Authors:** Isabel Fernandes Arroteia, Hans Christoph Bock, Andreas Schaumann, Valentina Pennacchietti, Ahmed El-Garci, Gesa Cohrs, Matthias Schulz, Friederike Knerlich-Lukoschus, Ulrich-Wilhelm Thomale

**Affiliations:** 1 Pediatric Neurosurgery, Charité Universitätsmedizin Berlin, Campus Virchow Klinikum, corporate member of Freie Universität Berlin and Humboldt-Universität zu Berlin, Augustenburger Platz 1, 13353 Berlin, Germany; 2 Neurosurgical Department, Göttingen University Hospital, Göttingen, Germany

**Keywords:** Infantile hydrocephalus, Ventriculoperitoneal shunt, Differential pressure adjustable valve, Gravitational adjustable valve

## Abstract

**Objective:**

Cerebrospinal fluid (CSF) drainage via a ventriculoperitoneal shunt remains to be the most common treatment for infantile hydrocephalus. Since head growth is particularly dynamic and susceptible to intracranial pressure changes during the first months of life, shunting can have a considerable impact. This study aimed to examine changes in head circumference and ventricular width and evaluate their interdependence in children who underwent shunting during infancy with either of two different adjustable valve regimens.

**Methods:**

We conducted a retrospective analysis on synchronized patient databases at two tertiary pediatric neurosurgical centers. One center implanted a differential pressure (DP) adjustable valve with a fixed gravitational unit (aDP_G_ group). The other center used an adjustable gravitational assistance (GA) valve combined with a fixed DP unit (aG_DP_ group). We included infants less than 2 years of age at shunt implantation with a follow-up time of 3 years, respectively. Patient demographics, head circumferences (*z*-scores), ventricular width and opening pressure settings of the shunt valves were collected and compared using statistical tests as appropriate.

**Results:**

A total of 155 infants (*n* = 78 in aDP_G_ group and *n* = 77 in the aG_DP_ group) could be included in this study. Changes in head circumference *z*-scores across follow-up time were similar in both groups (*p* = 0.99). In the aG_DP_ group, patients had more pronounced pathologically enlarged ventricles measured by fronto-occipital horn ratio (FOHR) at implantation compared to the aDP_G_ group (*p* = 0.037). At the end of follow-up, in both groups, the proportion of patients showing a normal FOHR (0.37 ± 0.026) increased significantly, while reduction in ventricular size over time was more pronounced in the aG_DP_ group (*p* < 0.01). The valve setting in the aG_DP_ group was significantly lower in standing and in lying position compared to the aDP_G_ group. Regression analysis revealed a significant association between the change in median FOHR and head circumference at the end of follow-up (slope (95%CI) = 3.46 (2.42–4.49), *R*^2^ = 0.22, *p* < 0.0001). Median change in FOHR, as well as head circumference at the end of follow-up, did correlate to valve settings at shunt implantation (p<0.01).

**Conclusions:**

The present study demonstrates a strong relation between changes in median FOHR and head circumference at the end of follow-up in shunted hydrocephalic infants. In addition, we identified a direct influence of initial valve setting on changes of ventricular width and head circumference at follow-up, underlining the importance of valve resistance for regulating CSF diversion and its anatomical consequences. Further investigations are needed to assess the influence of these parameters on long-term neurodevelopment and functional outcome.

## Introduction

The size of the cranium measured as head circumference is among the first physical screening features for intracranial pathologies during infancy. Deviations from the norm usually lead to further diagnostic measures. Atypical head sizes are indicative for microcephaly or macrocephaly and may be associated with neurodevelopmental impairment.

Infants diagnosed with hydrocephalus are commonly treated with ventriculoperitoneal shunts (VPS), which regulate CSF drainage. A wide variety of shunt valves is available on the market, including adjustable and non-adjustable options, some of which are accompanied with anti-siphon devices, gravitational units, or flow-regulation mechanisms. By physics, these diverse drainage mechanisms will somehow differently influence cerebrospinal fluid (CSF) flow dynamics. Achieving a near-physiological CSF flow rate through a shunt system may be relevant not only for promoting a balanced equilibrium of ICP and CSF-brain tissue ratio but also for preventing over- or underdrainage. Possible consequences of underdrainage are more often associated with acute or subacute clinical manifestations such as headache, nausea, and vomiting and other neurological symptoms mostly aggravating in lying position. In contrast, overdrainage may lead to more chronic complications such as microcephaly, hyperostosis, and slit-ventricle syndrome which may be less likely associated with clinical symptoms such as headache, irritability, and drowsiness more often seen in upright position or after physical activity. Previous studies indicate that adjustable shunt valves potentially reduce the incidence of these complications [1–4]. Close monitoring during follow-up is crucial to prevent both acute and chronic complications. As previously described, three indirect factors relevant to assess indirect measures for adequate CSF drainage are the evaluation of clinical symptoms as described above, measuring head circumference as percentile values, and evaluating ventricular size in follow-up cranial imaging. Ventricular width can be evaluated either by ultrasound in a state of open fontanelle or preferably by magnetic resonance imaging (MRI) at later stages.

Adjustable shunt valves offer the advantage of non-invasive pressure adjustments using strong magnets, allowing physicians to potentially fine-tune CSF drainage in order to react on either irregular head growth or differences in ventricular width over time. However, little is known about how specific valve pressure settings affect changes in head circumference and ventricular width usually measured as frontal-occipital horn ratio (FOHR). Moreover, data on the correlation between FOHR and head circumference remain limited [5].

This study aims to assess changes in head circumference and ventricular width over time and to explore the relationship between these two parameters in infants who underwent shunt implantation with two different valve adjustment regimens for treating hydrocephalus of various etiologies taken from a bi-center study.

### Methods

We conducted a retrospective study of infants diagnosed with hydrocephalus from two different tertiary pediatric neurosurgical centers, who underwent shunt implantation using either a differential pressure adjustable valve with a fixed gravitational assistance (aDP_G_ group) or an adjustable gravitational valve with a fixed differential pressure unit (aG_DP_ group). Patient data was collected from a synchronized in-house hydrocephalus registry [6] from each center established since 2009, completed by data acquired from digital patient charts. Ethical approval was given by local ethics committee from both participating institutions (EA2/132/17).

**Fig. 1:**
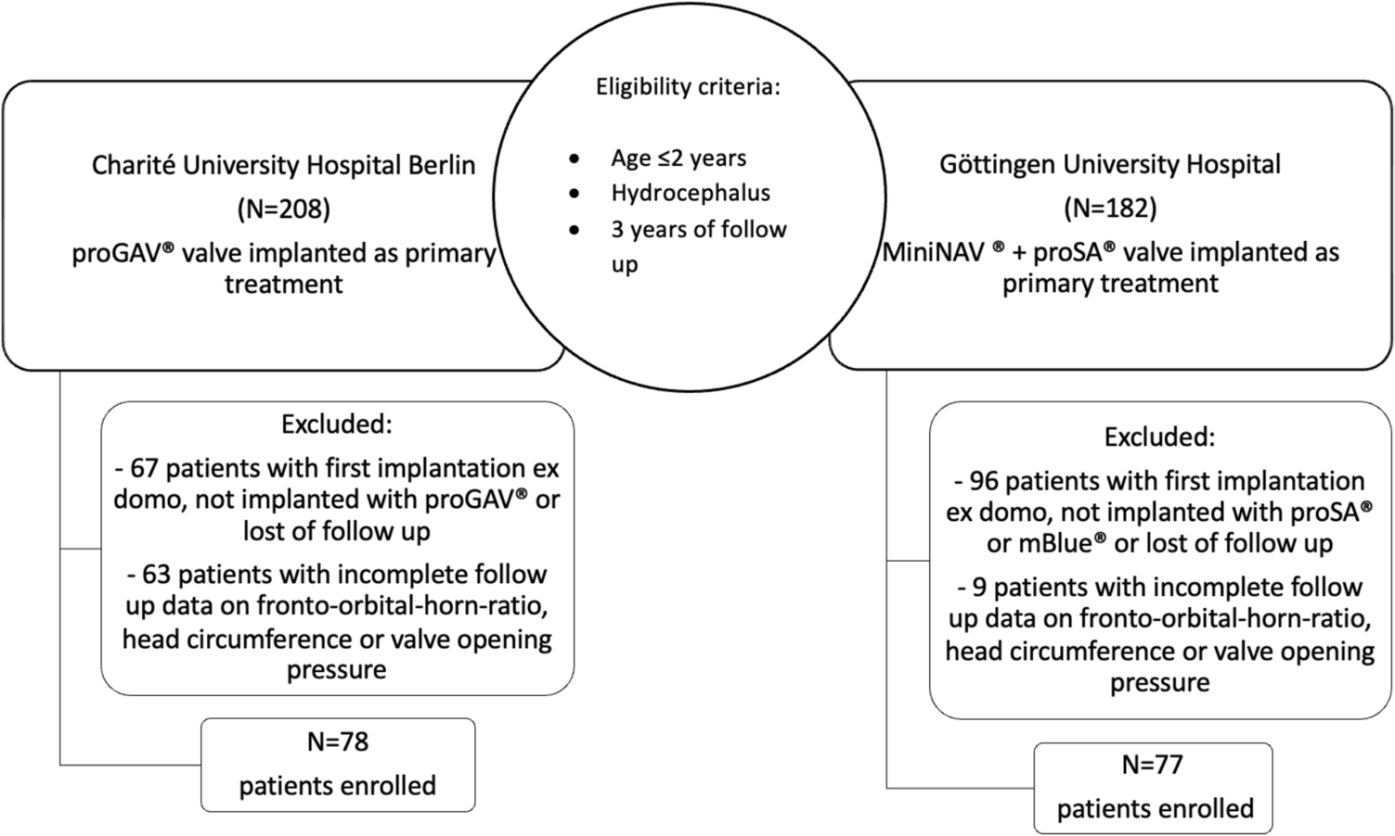
Flow diagram of patient inclusion process. Patients included in this study were diagnosed with shunt-dependent hydrocephalus and underwent primary shunt implantation before the age of 2 years. They were treated with either an adjustable gravitational valve combined with a fixed differential pressure unit (aG_DP_ group) or a differential pressure-adjustable valve combined with a fixed gravitational unit ( aDP_G_ group). To be eligible, patients had to have undergone their initial shunt implantation at one of the two participating medical centers, with a minimum follow-up of 3 years after surgery. Of the 390 patients screened, 155 met the inclusion criteria ( aG_DP_ group: *n* = 77; aDP_G_ group: *n* = 78)

The study included infants diagnosed with hydrocephalus who underwent ventriculo-peritoneal shunt implantation before 2 years of age and were followed up for 3 years post-implantation, respectively. In the aDP_G_ group, patients received a proGAV or proGAV2.0 valve system (Miethke-Aesculap, Potsdam/Tuttlingen, Germany) at Charité University Hospital Berlin between 2009 and 2017. Group aG_DP_ included patients who underwent implantation of a miniNav together with a proSA or a M.Blue valve (Miethke-Aesculap, Potsdam/Tuttlingen, Germany) at Göttingen University Hospital between 2009 and 2019. Only patients with complete data sets, including follow-up information on fronto-occipital-horn-ratio (FOHR), head circumference, and valve opening pressures before shunt implantation and at end of follow-up were included in the study (Fig. 1).

The proGAV consists of two combined units in one valve, an adjustable ball-in-cone-valve regulated by a rotor, which changes the tension of a spring acting on the valve mechanism, between 0 and 20 cmH_2_O resistance combined with a fixed gravitational unit (20 or 25 cmH_2_O) adding an additional resistance in upright body position [7]. The proGAV2.0 is a successor model to the proGAV valve, allowing for easier adjustability and showing a reduced internal lumen for CSF flow [8]. The proSA valve incorporates a similar rotor-based adjustability of the gravitational unit. Thus, the range of resistance being active in the upright position ranges from 0 to 40 cmH_2_O. The resistance in the horizontal position is determined by the miniNAV, a fixed differential pressure valve (5 cmH_2_O) [3]. The M.Blue valve is the successor model to the proSA, combining the miniNAV together with the proSA in one valve. All above-described valve types are resistant to external magnetic forces up to 3 Tesla and can be adjusted externally using transcutaneous pressure to release a break mechanism and apply a magnetic force to redirect the rotor position translating into a certain spring tension acting on the valve mechanism. Opening pressures were chosen according to the individual center preferences.

Patient data analysis included patient’s gender, prematurity, age at surgery, underlying disease, and previous surgeries before shunt implantation. Valve adjustments were documented at implantation and at the end of follow-up in both _G_ groups.

In radiological analysis, the ventricular width was measured using the fronto-occipital horn ratio (FOHR). That includes measurements of the bifrontal horn diameter (FHD), bioccipital horn diameter (OHD), and widest possible biparietal brain diameter (PD) at the level of the foramen of Monro and calculated as follows: (FHD + OHD)/2PD = FOHR [9]. As described in the literature, a FOHR value of 0.37 ± 0.026 was considered normal for our patient population [10]. FOHR measurements are reported to have a high interobserver reliability in pediatric patients [10, 11].

Head circumference (HC) was measured using a standardized method, involving three consecutive occipitofrontal measurements with a measuring tape, performed by experienced pediatric neurosurgeons. The mean of these measurements was calculated to minimize intrapersonal measurement error. HC data were collected both preoperatively and at approximately 3 years of follow-up. Preoperative HC *z*-scores and percentiles were calculated using open-source software PediTools [12] Fenton 2013 growth calculator for preterm infants, based on the Fenton Charts [13], and the WHO Anthro software [14] for term-born infants. For preterm infants implanted with a VPS after 50 weeks of gestational age—beyond the range of the Fenton Charts—age correction according to gestational age was applied, and *z*-scores and percentiles were calculated using WHO growth charts [15] through the Anthro software. All postoperative *z*-scores and HC percentiles were calculated using WHO Anthro Software also applying possible age-correction for preterm-born infants.

### Statistical analysis

Data were extracted from the institutional hydrocephalus registry [6] (Filemaker, Claris, Cupertino, CA, USA) into an Excel data sheet (Microsoft Office, Alberquerque, NY, USA). Statistical analysis was conducted using IBM SPSS® software (IBM, Armonk, NY, USA). Graphs were generated using GraphPad Prism (GraphPad Software Inc., San Diego, CA, USA). Patient demographic data was given as mean ± standard deviation or median and interquartile range. Chi-square test was performed to compare *z*-score and FOHR distribution. Simple linear regression analysis was performed on head circumference, FOHR and valve opening pressure data. A *p*-value of less than 0.05 was considered statistically significant.

## Results

### Patient characteristics

A total of 78 patients could be included in the aDP_G_ group, while 77 patients were included in the aG_DP_ group. The rate of patients implanted under the age of 1 year was similar in both groups (93.6% vs. 93.5%, *p* = 0.983). However, the aG_DP_ group included significantly more preterm infants (46.2% vs. 66.2%, *p* = 0.012). The median age at shunt implantation was similar for preterm-born infants in both groups with 43.4 weeks (37.1–62.0) in the aDP_G_ group (*N* = 36) versus 39.7 weeks (36.9–47.4) in the aG_DP_ group (*N* = 51; *p* = 0.352), but significantly higher in term-born infants of the aG_DP_ group with 2.2 months (1.2–7.2; *N* = 26) compared to the aDP_G_ group with 0.8 months (0.4–2.2; *N* = 42; *p* = 0.017). Median time span from birth to implantation was higher in the aG_DP_ group compared to aDP_G_ group (12.6 weeks, IQR 6–23.9 versus 8.3 weeks, IQR 2.4–18.7; *p* = 0.051). Mean FOHR at time of shunt-implantation was significantly higher in the aG_DP_ group (0.59 ± 0.10 vs. 0.55 ± 0.11; *p* = 0.011).

The etiology of hydrocephalus differed in both groups, with a higher rate of posthemorrhagic hydrocephalus in the aG_DP_ group (54.5% vs. 35.4% in the aDP_G_ group; *p* = 0.016) while malformative hydrocephalus was more represented in the aDP_G_ group (60.8% vs. 44.2% in the aG_DP_ group; *p* = 0.038). Treatment strategy before shunt implantation differed in both centers, with overall a higher rate of interventions applied to the patients in the aG_DP_ group (68.8% vs. 43% in the aDP_G_ group; *p* = 0.001). Patients included in the aDP_G_ group underwent more frequently a neuroendoscopic lavage before shunt implantation (20.3% vs. 3.9% in the aG_DP_ group; *p* = 0.002), while patients included in the aG_DP_ group were more likely to be subjected to the implantation of a ventricular access device (50.6% vs. 25.3% in the aDP_G_ group, *p* = 0.001) (Table 1).

**Table 1:** Patient characteristics

Characteristics	aDP_G_	aG_DP_	*p*-values
**Age of preterm babies at first implantation** (GA* in weeks; median (IQR))	43.4 (37.1–62.0) (*N* = 36)	39.7 (36.9–47.4) (*N* = 51)	*p* = 0.352
**Age of term babies at first implantation** (months; median (IQR))	0.8 (0.4–2.3) (*N* = 42)	2.2 (1.2–7.2) (*N* = 26)	***p* = 0.017**
**Sex ratio (f/m)**	30/48	27/50	*p* = 0.661
**Time span from birth to implantation** (weeks; median (IQR))	8.2 (2.4–18.9) (*N* = 78)	12.6 (6–23.9) (*N* = 77)	*p* = 0.055
**Rate of patients ≤ 1 year at shunt implantation**	93.6% (*N* = 73/78)	93.5% (*N* = 72/77)	*p* = 0.983
**Rate of preterm infants**	46.2% (*N* = 36/78)	66.2% (*N* = 51/77)	***p* = 0.012**
**Etiology**	(*N* = 78)	(*N* = 77)	
Posthemorrhagic	34.6%	54.5%	***p* = 0.013**
Postinfectious	3.8%	1.3%	*p* = 0.317
Malformative	61.5%	44.2%	***p*= 0.030**
Associated to cranial or spinal expansive lesions	1.3%	1.3%	*p* = 0.993
**Pre-shunt operations**	(*N* = 78) 43.6%	(*N* = 77) 68.8%	***p*= 0.002**
NEL	20.5%	3.9%	***p*= 0.002**
VAD	25.6%	50.6%	***p*= 0.001**
EVD	3.8%	2.6%	*p* = 0.660
Septostomie	3.8%	2.6%	*p* = 0.660
ETV	12.8%	1.3%	*p* = 0.005
Other	5.1%	20.8%	***p*= 0.004**

*aDP*_*G*_, patient group implanted with an adjustable differential pressure valve together with a fixed pressure gravitational unit

*aG*_*DP*_, patient group implanted with an adjustable gravitational valve together with a fixed differential pressure unit

*IQR*, interquartile range; GA*, gestational age; *f*, female; *m*, male; *NEL*, neuroendoscopic lavage; *VAD*, ventricular access device; *ETV*, endoscopic third ventriculostomy; *other*, tumor removal, MMC coverage, Chiari decompression

*SD*, standard deviation, p p-values (statistical significance at p<0.05; bold entries)

### Preoperative distribution and changes in head circumference z-scores

**Fig. 2:**
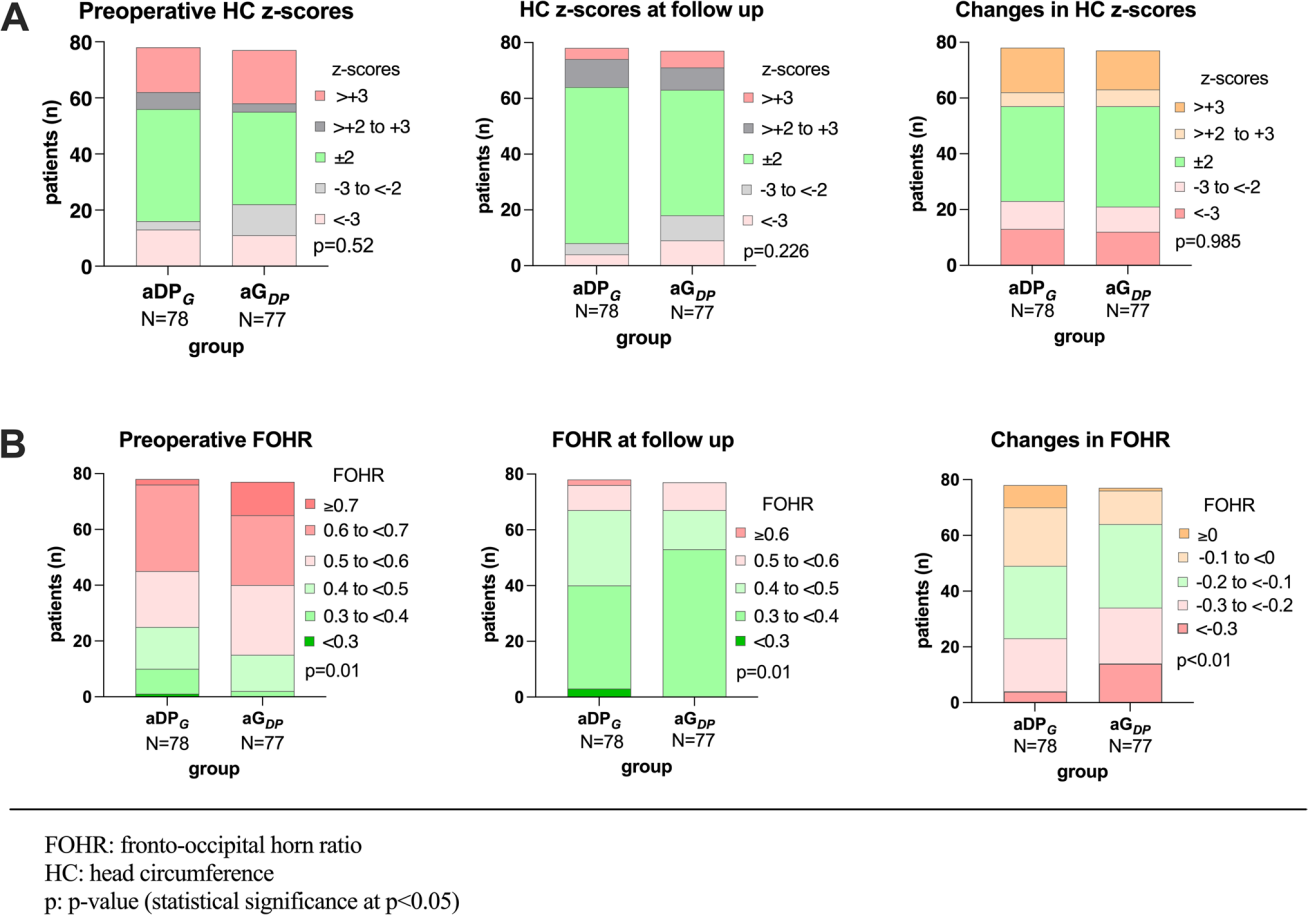
Changes in head circumference and FOHR over follow-up time. FOHR, fronto-occipital horn ratio; HC, head circumference. **a** No significant difference was observed in the preoperative distribution of head circumference *z*-scores between the two groups (*p* = 0.52) or after 3 years of follow-up (*p* = 0.226). Additionally, changes in head circumference *z*-scores over time were highly similar in both groups (*p* = 0.985). **b** At implantation, the aG_DP_ group had a higher proportion of patients with pathologically enlarged ventricles (*p* = 0.037). By the 3-year follow-up, both groups showed a significant increase in the proportion of patients with normal or decreased FOHR. The aG_DP_ group had a greater proportion of patients achieving a normal FOHR (*p* < 0.0001) and experienced more pronounced ventricular size reduction, with more patients showing a decrease of over 0.2 and 0.3 in FOHR (*p* < 0.01)

No significant difference could be shown in the preoperative distribution of head circumference *z*-scores in both groups (*p* = 0.52), neither after 3 years of follow-up (*p* = 0.226). Changes in head circumference *z*-scores across follow-up time could be shown to be very similar in both groups (*p* = 0.985; Fig. 2a). Similarly, there were no notable differences in median (IQR) head circumference at implantation (aDP_G_ 0.34 (−3.85–4.53) vs. aG_DP_ −0.01 (−5.65–5.63), *p* = 0.878), at the end of follow-up (aDP_G_ 0.4 (−2.19–2.99) vs. aG_DP_ −0.15 (−3.71–3.41), *p* = 0.367), or in the change of head circumference *z*-scores over the observation period (aDP_G_ −0.19 (−5.24–4.86) vs. aG_DP_ −0.33 (−4.7–4.04), *p* = 0.72, Table 2).

**Table 2:** FOHR, head circumference *z*-scores, and valve opening pressures at implantation and end-of-follow-up

	At implantation			At end-of-follow-up			Δ		
Variables	aDP_*G*_(*N* = 78)	aG_*DP*_(*N* = 77)	*p*	aDP_*G*_(*N* = 78)	aG_*DP*_(*N* = 77)	*p*	aDP_*G*_(*N* = 78)	aG_*DP*_(*N* = 77)	*p*
FOHR median (IQR)	0.57 (0.42–0.72)	0.58 (0.42–0.74)	**0.034**	0.39 (0.24–0.54)	0.37 (0.32–0.42)	0.870	−0.15 (−0.31–0.01)	−0.18 (−0.33–(−0.03))	**0.002**
HC *z*-score median (IQR)	0.34 (−3.85–4.53)	−0.01 (−5.65–5.63)	0.878	0.4 (−2.19–2.99)	−0.15 (−3.71–3.41)	0.367	−0.19 (−5.24–4.86)	−0.33 (−4.7–4.04)	0.720
Valve setting in standing position (cmH_2_O; median (IQR))	29 (27–31)	25 (0)	** < 0.001**	30 (24–36)	25 (20–25)	** < 0.001**	0 (0–5)	0 (0)	**0.007**
Valve setting in lying position (cmH_2_O; median (IQR))	9 (7–11)	5 (0)	** < 0.001**	10 (7–13)	5 (0)	** < 0.001**	0 (0–3)	0 (0)	**0.007**

*aDP*_*G*_, patient group implanted with an adjustable differential pressure valve together with a fixed pressure gravitational unit

*aG*_*DP*_, patient group implanted with an adjustable gravitational valve together with a fixed differential pressure unit

IQR: interquartile range; GA*, gestational age; *f*, female; *m*, male; *NEL*, neuroendoscopic lavage; *VAD*, ventricular access device; *ETV*, endoscopic third ventriculostomy; *other*, tumor removal, MMC coverage, Chiari decompression

*SD*, standard deviation

Δ, difference between the preoperative and follow-up values

*FOHR*, fronto-occipital horn ratio

*HC*, head circumference

*p*, *p*-values (statistical significance at *p* < 0.05; bold entries)

### Preoperative distribution and changes in FOHR

In the aG_DP_ group, the majority of patients (97.4%) presented with significantly enlarged ventricles at implantation, as indicated by an elevated FOHR, while only a small proportion (2.6%) exhibited a normal preoperative FOHR. In contrast, a higher proportion of patients in the aDP_G_ group (12.8%) had a normal or below-normal FOHR at implantation (*p* = 0.037). By the end of follow-up, both groups showed an increase in the proportion of patients with a FOHR below 0.4. At the end of follow-up, there was a significant difference in FOHR distribution between the groups (*p* = 0.01), with a larger proportion in the aG_DP_ group exhibiting a FOHR under 0.4. Moreover, the distribution of changes in FOHR over follow-up period differed significantly between both groups (*p* < 0.01), with a more pronounced reduction in ventricular width in the aG_DP_ group. This group had a higher proportion of patients experiencing a FOHR decrease of more than 0.2 or even 0.3 (Fig. 2b). A significant difference in median (IQR) FOHR was observed at implantation (aDP_G_ 0.57 (0.42–0.72) vs. aG_DP_ 0.58 (0.42–0.74), *p* = 0.034), but not at the end of follow-up (aDP_G_ 0.39 (0.24–0.54) vs. aG_DP_ 0.37 (0.32–0.42), *p* = 0.870), resulting from a significant difference in median (IQR) decrease of FOHR over the observation period (aDP_G_ −0.15 (−0.31–0.01) vs. aG_DP_ −0.18 (−0.33–(−0.03), *p* = 0.002, Table 2).

### Regression analysis of FOHR and head circumference z-scores

**Fig. 3:**
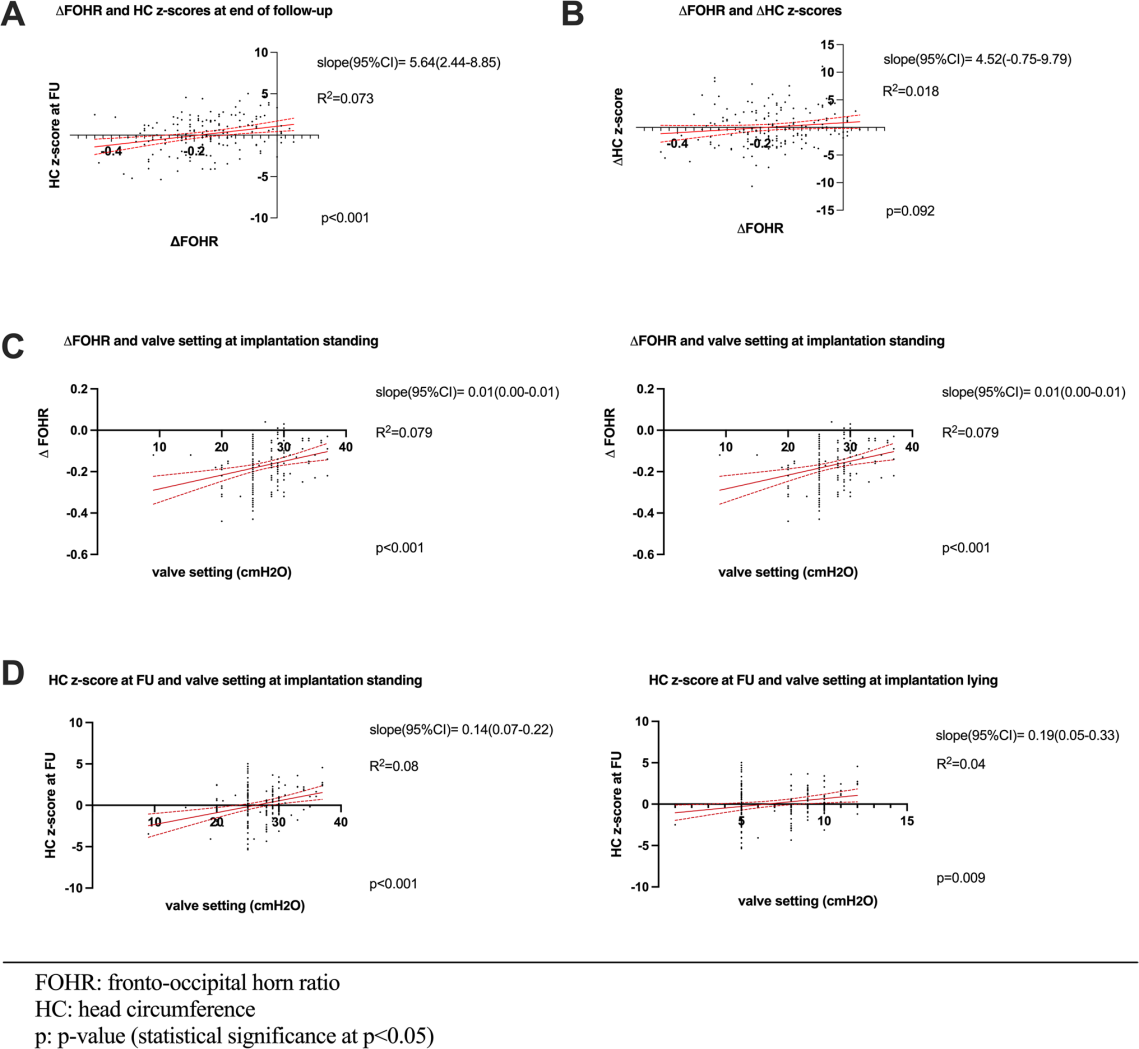
Regression analysis of head circumference *z*-scores and FOHR (all patients). FOHR, fronto-occipital horn ratio; HC, head circumference. **a** Regression analysis showed a significant association between the change in median FOHR and head circumference at the end of follow-up. **b** No significant relationship between the changes in median FOHR and changes in median head circumference z-scores over 3 years of follow-up was observed. **c**, **d** Median change in FOHR, as well as head circumference *z*-scores at the end of follow-up showed to correlate to the adjusted valve resistance at shunt implantation, both in standing and lying position

Regression analysis revealed a significant association between the change in median FOHR and head circumference at the end of follow-up (slope (95%CI) = 5.64 (2.44–8.85), *R*^2^ = 0.073, *p* < 0.001). However, no significant relationship between the changes in median FOHR and changes in median head circumference *z*-scores over 3 years of follow-up was observed (slope (95%CI) = 4.52 (−0.75–9.79), *R*^2^ = 0.018, *p* = 0.092, Fig. 3).

### Analysis of correlations between FOHR, head circumference z-scores and valve resistance settings

Correlation between the change in median FOHR and head circumference *z*-scores at the end of follow-up was shown to be significant (*r* (95%CI) = 0.27 (0.12–0.14), *R* = 0.073, *p* < 0.001). However, no significant correlation was found between the median variation of FOHR and the median variation in head circumference *z*-scores over 3 years of follow-up (*r* (95%CI) = 0.14 (−0.02–0.29), *R*^2^ = 0.018, *p* = 0.092). Median change in FOHR, as well as head circumference *z*-scores at the end of follow-up showed to correlate to the adjusted valve resistance at shunt implantation, both in standing (*r* (95%CI) = 0.28 (0.13–0.42), *R*^2^ = 0.079, *p* < 0.001 and *r* (95%CI) = 0.29 (0.14–0.43), *R*^2^ = 0.083, *p* < 0.001) and lying position (*r* (95%CI) = 0.25 (0.09–0.39), *R*^2^ = 0.061, *p* = 0.0018 and *r* (95%CI) = 0.21 (0.05–0.35), *R*^2^ = 0.043, *p* = 0.009, illustrated by regression analysis in Fig. 3c and d). Valve resistance was set higher in the aDP_G_ group compared to the aG_DP_ group at implantation (standing; aDP_G_ 29 (27–31) vs. aG_DP_ 25 (0), *p* < 0.001, lying; aDP_G_ 9 (7–11) vs. aG_DP_ 5 (0), *p* < 0.001)) and at the end of follow-up (standing; aDP_G_ 30 (24–36) vs. aG_DP_ 25 (20–25), *p* < 0.001, lying; aDP_G_ 10 (7–13) vs. aG_DP_ 5 (0), *p* < 0.001), Table 2).

## Discussion

With the current study, we investigated the baseline and development of the ventricular width and head circumference in relation to the valve settings in two cohorts of infants with hydrocephalus initially treated with VPS and gravitational assisted valves during 3 years of follow-up. We were able to show that lower settings in the aG_DP_ group did lead to more extended reduction in ventricular width. In addition, there was a significant correlation between the valve setting with both outcome values of the reduction of ventricular width as well as the overall head circumference at the end of follow-up. To our knowledge, this is the first study primarily aimed at assessing the influence of early infancy shunting on both ventricular expansion and skull development, as measured by head circumference.

### Relationship between ventricular width and head circumference

Venkatraman et al. recently investigated the ability of the head circumference-to-ventricular size ratio to predict shunting rates in premature infants with intraventricular hemorrhage (IVH) and demonstrated that these ratios differed significantly between high-grade and low-grade IVH. Since values fluctuated over time, measurements of head circumference do not change proportionally to ventricular size [5]. When comparing the two shunt valve regimens used in our study, both cohorts showed a similar distribution of head circumference *z*-scores at follow-up, as well as comparable changes in head circumference over time, even though other patterns were observed for ventricular width . At implantation, infants in the aG_DP_ group had significantly wider ventricles. However, at the end of follow-up, a greater proportion of patients in the aG_DP_ group had achieved a FOHR of 0.37 ± 0.026, a value reported as normal in healthy children [10]. While the mean FOHR were similar in both groups, the distribution of FOHR differed at the end of follow-up and ventricular width reduction was more pronounced in the aG_DP_ group, with significantly more patients experiencing a FOHR decrease of over 10% or even 20%. In contrast, there was no significant differences neither in mean values nor in the distribution in head circumference *z*-scores. A similar trend has been reported in a study comparing craniometrics after endoscopic thirdventriculo-cisternostomy with choroid plexus cauterization (ETV/CPC) versus VPS implantation, showing significantly smaller ventricular widths in the VPS group, yet no significant difference in postoperative head circumference *z*-scores between the two groups at 6 months [16]. Comparable findings were reported in a post hoc analysis of the International Infant Hydrocephalus Study (IIHS) [17] comparing craniometrics after ETV versus VPS implantation in infants at 12 months (0.52 vs 0.44; p = 0.002) and 3 years (0.46 vs 0.38; p = 0.03) of follow-up [18]. Ingram et al. similarly found no correlation between Evans index (EI) or changes in EI and head circumference, concluding that those two parameters are not necessary surrogates for one another [19]. While looking at more detail in our data, our study identified a significant association between 3-year head circumference *z*-scores and the median change in FOHR and a tendency of correlation of changes in *z*-scores and changes in FOHR. Thus, our data suggest a relationship between the effect of CSF drainage on ventricular width and changes in head circumference over long-term follow-up.

### Association of ventricular width and head size with valve opening pressure at implantation

Our study found a significant association between valve opening pressure at implantation and ventricular width reduction over time, as well as between initial valve opening pressure and the head circumference at the end of follow-up. Since we were looking only at a limited follow-up time of 3 years, the results are still not clear but it might be speculated that lower VP shunt settings are actually causing lower ventricular size and might impair normal head growth and development. In both groups, the valve settings at implantation differed significantly; however, clear differences emerged in 3-year ventricular width , but not necessarily in head circumference. Both valve types incorporate a gravitational assistance to prevent severe overdrainage which seems to be effective especially in establishing normal head growth over time. Further comparisons with treatment strategies using simple differential pressure valves would be important to potentially look at even higher discrepancies in utilized valve resistances. It also raises the question of how far adjustability of the valve resistance can be used as therapeutical measure to establish physiological like anatomic development of the ventricles and the head size in shunted patients. However, despite the increasing adoption of adjustable shunt valves, evidence-based adjustment protocols still remain lacking. Previously, we discussed about applying the valve setting on the basis of three factors: firstly, clinical symptoms of overdrainage; secondly, changes in head circumference in terms of crossing percentile values; and thirdly, relevant changes of ventricular width in regular follow-up imaging. These three factors might guide the treating team to adapt the CSF drainage via the shunt by adjusting the valve resistance setting accordingly. Still, establishing a standardized and reliable protocol warrants more scientific evidence. Based onour current data, we are able to define first steps towards this direction. The long-term goal is to significantly improve treatment quality by reducing uncertainty in the use of adjustable valves and by establishing more physiological CSF drainage via a VPS, even under the constantly changing conditions of a growing and developing child.

### Relation of initial ventricular width and long-term outcome

In our study, we evaluated outcomes over a 3-year follow-up period, aware that longer observation is needed to fully assess how CSF drainage influences long-term cranial development and whether additional valve adjustments are required once ventricular normalization is achieved. tT Treating affected infants at an earlier stage of ventricular dilatation may further improve neurological outcome while preserving brain parenchyma. The Early vs. Late Ventricular Intervention Study (ELVIS) [20] demonstrated that early CSF punctures resulted in better outcomes compared to delayed intervention in preterms with intraventricular hemorrhage (IVH), with a post hoc analysis of ELVIS further showing that patients who received later interventions exhibited larger ventricular volumes and more brain injury at follow-up [21]. Lai et al. showed greater ventricular dilation in preterm infants with IVH to be independently associated with worse school-aged functional outcome in Grade III and IV IVH, regardless of neurosurgical intervention (*p* < 0.01), with a maximum FOHR of 0.61 being shown to be a predictor of moderate-severe impairment (AUC 75%, 95% CI 62–87%) [22]. Pauturu et al. similarly showed in preterms with PHVD, a smaller cumulative ventricular size from birth to permanent CSF diversion to be associated with larger right hippocampal volumes on term-equivalent MRI (Pearson’s *r* = −0.483, *p* = 0.014) and improved cognitive (*r* = −0.711, *p* = 0.001), motor (*r* = −0.675, *p* = 0.003), and language (*r* = −0.618, *p* = 0.011) outcomes at 2 years of age [23]. This findings might be due to the fact that pressure-induced ventricular dilation might decrease regional cerebral oxygen saturation, which could be reversed after ventricular decompression as shown in children with PHVD [24], thereby avoiding additional brain damage. However, persistent larger than normal ventricular size after hydrocephalus treatment did not show to be associated to additional white matter injury or worse neurocognitive deficits [25]. Further investigations will certainly be necessary to elucidate the influence of ventricular width and also brain parenchymal volume changes due to different strategies of CSF drainage patterns on neurocognitive development.

### Limitations

This study´s retrospective nature introduces the possibility of selection bias, as only patients with complete datasets were included. Additionally, neurocognitive outcomes were not assessed, preventing us from evaluating any potential relationships between head circumference, ventricular width, and long-term cognitive development. To generalize these findings, they must be confirmed in a multicenter prospective study, including patients treated with various shunt valve types.

Nevertheless, this study is the first to demonstrate a statistically significant relationship between FOHR reduction and head circumference at 3-year follow-up. This underscores the need for further research to explore additional factors influencing this relationship and its impact on clinical outcomes in specific pediatric populations. A longer follow-up period is also necessary to assess whether more pronounced CSF drainage influences long-term cranial development and whether further valve adjustments are required once ventricular normalization is achieved. As assessment of ventricular width in children presenting closed fontanelles is mostly performed by MRI, this raises the question of whether ventricular volumetric studies could add some value to the radiological assessment of these children, compared to FOHR used in our study. Interestingly, Cizmeci et al. were able to show that FOHR presents good correlation with MRI based ventricular volumetric measurements (*r* = 9.62; *p* < 0.001) [21] As similar results were also described by additional studies [11, 26, 27], that will be an important direction of further research.

## Conclusions

The results of this study indicate that the median decrease in FOHR over time seems to be a relevant predictor of head circumference development at 3-year follow-up. This underscores the importance of regular monitoring of both head circumference and FOHR during clinical follow-up appointments for these children. In the context of adjustable shunt valves, understanding ventricular width dynamics may offer clinicians a valuable tool for optimizing long-term cranial development by actively adjusting CSF drainage through modifications of valve resistance. Thereby, clinicians can potentially influence the trajectory of head circumference growth, ensuring better overall management of pediatric hydrocephalus. Future prospective studies should further investigate the long-term impact of CSF drainage strategies on ventricular width, head circumference, and their impact on neurodevelopmental outcomes, helping refine treatment approaches and improve patient care.

## Data Availability

The datasets generated and/or analyzed during the current study are not publicly available due to institutional data protection policies but are available from the corresponding author on reasonable request.
